# A Bayesian variable selection procedure to rank overlapping gene sets

**DOI:** 10.1186/1471-2105-13-73

**Published:** 2012-05-03

**Authors:** Axel Skarman, Mohammad Shariati, Luc Jans, Li Jiang, Peter Sørensen

**Affiliations:** 1 Department of Molecular Biology and Genetics, Aarhus University, Blichers Allé 20, PO Box 50, Aarhus, Tjele DK-8830, Denmark; 2 Department of Animal Science, Ferdowsi University of Mashhad, Mashhad, 91775, Iran

**Keywords:** Bayesian variable selection, Gene set, Overlap

## Abstract

**Background:**

Genome-wide expression profiling using microarrays or sequence-based technologies allows us to identify genes and genetic pathways whose expression patterns influence complex traits. Different methods to prioritize gene sets, such as the genes in a given molecular pathway, have been described. In many cases, these methods test one gene set at a time, and therefore do not consider overlaps among the pathways. Here, we present a Bayesian variable selection method to prioritize gene sets that overcomes this limitation by considering all gene sets simultaneously. We applied Bayesian variable selection to differential expression to prioritize the molecular and genetic pathways involved in the responses to *Escherichia coli* infection in Danish Holstein cows.

**Results:**

We used a Bayesian variable selection method to prioritize Kyoto Encyclopedia of Genes and Genomes pathways. We used our data to study how the variable selection method was affected by overlaps among the pathways. In addition, we compared our approach to another that ignores the overlaps, and studied the differences in the prioritization. The variable selection method was robust to a change in prior probability and stable given a limited number of observations.

**Conclusions:**

Bayesian variable selection is a useful way to prioritize gene sets while considering their overlaps. Ignoring the overlaps gives different and possibly misleading results. Additional procedures may be needed in cases of highly overlapping pathways that are hard to prioritize.

## Background

Genome-wide expression profiling using microarrays or sequence-based technologies allows us to identify genes and genetic pathways whose expression patterns influence complex traits. It is likely that many phenotypic differences are manifested by small but consistent expression changes in a set of genes (e.g. biological pathways, complexes or modules). Therefore statistical methods have been developed to capture changes in the expression of pre-defined sets of genes. These gene set approaches are complementary to analyses at the single-gene level and represent powerful tools to dissect the complex changes in gene expression that underlie phenotypic traits [1].

Gene set approaches are based on sets of genes typically defined based on prior biological knowledge, such as genes belonging to the same molecular pathway (Kyoto Encyclopedia of Genes and Genomes (KEGG)) [2] or genes encoding proteins with similar functions (*e.g.*, Gene Ontology (GO)) [3]. One potential problem is that genes can exist in many gene sets and the level of overlap can be substantial. This is ignored in statistical analyses where the gene sets are analyzed individually, which can cause difficulty in interpreting the results, because one cannot determine from the available data which one of the gene sets is more responsible for the effect. In the most extreme case, there could be one or more that are identical. In these cases it is impossible to determine which is responsible for the effect [4].

One powerful gene set approach is Gene Set Enrichment Analysis (GSEA) [1,5]. GSEA aggregates the per-gene statistics within a gene set, thus making it possible to detect situations where all the genes in a predefined set change in a small but coordinated way. GSEA can be implemented in a manner similar to a linear regression modeling approach that consists of three components: the incidence matrix linking genes to the gene set; the per-gene statistic vector, *e.g.*, the *t*-statistic, and a per-set summing function. In this way, a large number of gene sets and overlapping gene sets can be viewed as a linear regression with a large number of highly collinear regression variables. This is a typical combinatorial and model selection problem. One way of handling this problem has been to use linear modeling to select a model [4]. This could, for example, be achieved using Akaike’s Information Criterion (AIC) [6]. However, the optimizations according to this criterion should in principle compare all 2^*p*^ possible models (where *P* is the number of gene sets). This becomes computationally demanding as the number of gene sets increases.

Another way to consider the overlaps is the TopGO approach [7]. This takes into account the hierarchical structure of the gene sets among GO terms. However, this method is limited to overlaps that appear because of this hierarchical structure. The DAVID gene classification tool is another method that takes account of overlaps [8,9]. In this tool, the more highly overlapping gene sets are organized as groups [10]. This method analyzes these groups of gene sets instead of the individual gene sets, and makes it possible to score the groups according to the scores of the member gene sets.

In this study we present a gene set approach based on the Bayesian variable selection method, known as Stochastic Search Variable Selection (SSVS) [11]. The Markov chains underlying such Bayesian methods are efficient at handling combinatorial problems such as model selection. This approach can deal with a large number of gene sets and considers the overlaps among the gene sets. Instead of first finding the gene sets with significant effects and thereafter assessing their overlaps and correcting for the correlations among them, this approach should accomplish this process in just one step. The focus now is to investigate how the variable selection procedure analyzes the overlaps among gene sets and how this affects the prioritization of gene sets. Here, we demonstrate the use of this novel gene set approach in a genome-wide expression study of *Escherichia coli*-induced mastitis (udder infection) in dairy cattle.

## Methods

The gene set approach presented in this study is based on a linear model to identify and prioritize KEGG pathways whose expression levels are associated with bovine mastitis.

### Gene sets defined by KEGG pathways

KEGG pathways were used as gene sets. These pathways are a collection of high-quality molecular interaction and reaction networks representing the current knowledge of many important biological processes. The use of KEGG pathways as gene sets illustrates situations where the number of gene sets is relatively large and where the level of overlap among the gene sets is substantial. We used 196 KEGG pathways and 3130 bovine Entrez gene identifiers. Only KEGG pathways containing more than four genes were included. The number of genes in a set ranged from five to 793 (‘Metabolic Pathways’). The number of occurrences of the same gene across pathways ranged from one to 41. The KEGG pathways were taken from the KEGG database using the genome-wide annotations of bovine Entrez gene identifiers.

Scaling up the analysis to higher dimensions and to cases with increased overlap, as when GO is used to define gene sets, was not expected to be problematic. The current analysis uses little memory and computing time, and both will scale-up linearly in these Bayesian implementations. With higher overlaps among gene sets, worse mixing for gene effects among sets can be expected, probably requiring some increase in the Markov chain Monte Carlo chain length to obtain accurate estimates.

### Linear model

The gene set approach is based on a linear model that in matrix notation can be written as

(1) z=μ+Xβ+e,

where **z** is the per-gene statistic (*e.g.*, *t*-statistic), which is a measure of the association between the individual genes and the trait phenotype; μ is the general mean; **X** is an incidence matrix linking genes to the gene set and the per-gene statistic **z**. The residuals **e** are assumed to be independent and identically distributed according to **e** ~ N (0, **Iσ**^2^). The elements of the incidence matrix have a non-zero value if the gene belongs to the gene set and zero otherwise. To account for direction of the expression changes we used a −1 for genes that are down regulated and a 1 for those that are up regulated. Each row of the incidence matrix corresponds to a gene and each column to a gene set. For this study, the full incidence matrix had 3130 rows (corresponding to the total number of genes) and 196 columns (corresponding to the total number of KEGG pathways) ( Additional file 1); **β** is the regression coefficient that is the summary statistic for each pathway.

### ANOVA

Analysis of Variance (ANOVA) can be used to identify gene sets that explain a larger proportion of the variance in **z**, using the least squares technique. This can be achieved by fitting one gene set at a time and ignoring the overlap among gene sets [4]. To account for the overlap it is necessary to fit multiple gene sets simultaneously. The total number of models possible to create from this is 2^*p*^, where *p* is the number of gene sets. This could become computationally challenging for model selection based on comparisons of all possible models such as, for example, AIC [6]. Thus ANOVA-based testing of one gene set at a time was used as the reference method in comparison to the Bayesian variable selection method described in detail below. The test was corrected for multiple testing using the false discovery rate method of Benjamini and Yekutieli [12].

### Bayesian variable selection method

The Bayesian variable selection method was built on the linear model described above. It considers all gene sets simultaneously to identify the best multi-gene-set model to explain the data. The large number of gene sets is handled by SSVS [11]. The latent variable, *γ*_*i*_, is used, which can take the values one or zero to indicate whether a gene set (KEGG pathway) contributes to differences in the expression *t*-statistic. The distribution of the regression coefficient *β*_*i*_ is conditioned on *γ*_*i*_ by

(2) $$\beta_{i}|\gamma_{i}\sim \left(1-\gamma_{i}\right)N\left(0,\tau_{0}^{2}\right)+\gamma_{i}N\left(0,c^{2}\tau_{1}^{2}\right)\text{.}$$

The model uses a small prior probability for *γ*_*i*_ to be 1, and $\tau_{0}^{2}$ is chosen to be a small number while $\tau_{1}^{2}$is conditioned to be larger than$\tau_{0}^{2}$ and is estimated from the data, which has the effect that most regression coefficients *β*_*i*_ are (very) small. *γ*_*i*_ = 1 indicates that KEGG pathway *i* is present in the model and *γ*_*i*_ = 0 indicates that KEGG pathway *i* is absent from the model. The details of the Bayesian analysis are given in Additional file 2.

Both the dimension problem and the problem of comparing all 2^*p*^ models are countered by the use of Gibbs sampling [13]. The Gibbs sampler generates sequences of Markov chain Monte Carlo samples of the latent variables that converge rapidly to the posterior distributions of the latent variables. The Gibbs sampler also generates a sequence of *β* values and residual standard deviations σ as well as the latent variables *γ*. These variables are dependent on each other. From the posterior probabilities of the indicator variables Bayes Factors were computed as the posterior odds divided by the prior odds for including a predictor in the model [14].

In this way, the computational complexity would be O(rnm) where *r* is the number of iterations of the simulation, *n* is the total number of genes in all the KEGG pathways, and *m* is the number of KEGG pathways. It is clear that the number of possible models and the dimensional problem do not drastically affect the computation time. This is mainly because computationally demanding matrix multiplications are avoided. The combinatorial problem is also reduced, because the Markov chain converges to the posterior distribution of the model probabilities.

Ultimately, this method would be reasonably fast but still able to account for overlaps among the gene sets. The identification of gene sets is based on the average *t*-statistic for the genes in the set, and therefore there is no principle relationship between the size of the gene set and the chance of selecting a gene set for the model. The variable selection method was implemented in the software package iBay (http://www.bayz.biz) [15].

### Genome-wide expression data in relation to bovine mastitis

We demonstrated our approach using data from a genome-wide expression study of mastitis in dairy cattle [16]. The aim was to identify the global changes in mammary gland gene expression associated with bovine *E. coli*-induced mastitis during the acute and chronic stage of the infection in early lactating dairy cows. Sixteen healthy Danish Holstein-Friesian cows were challenged intra-mammarily with *E. coli* 4 to 6 weeks after parturition. Udder tissue biopsies were collected ante-mortem from dairy cows during the acute (24 h) and the chronic (192 h) stages of the *E. coli* infection. Further experimental details can be found in the original publication [16].

Gene expression was measured using the Bovine Genome array (Affymetrix, Santa Clara, CA). The array contained 24128 probe sets that represented 11030 Entrez genes. The Bovine Genome Array annotation available from the NetAffx™ Analysis Centre (Bovine.na29.annot.csv) was used as well as bovine.db (version 2.3.5) in Bioconductor [17]. In total, 3130 Entrez genes were assigned to KEGG pathways using the package org.Bt.eg.db (version 2.3.5).

The primary gene expression data were analyzed using R (version 2.10.1) [18]. Normalization of the expression values for the udder was performed using the Robust Multi-array Average algorithm implemented in the Affy package (version 1.24.2) [19]. Differential expression of individual genes was computed using linear modeling and empirical Bayes methods, which were implemented in the R package Limma (version 3.2.2) [20]. The linear models allowed for changes in the time-points. The time-points were 24 h and 192 h. The contrast used was 24 h *versus* 192 h post-infection. A modified *t*-value was computed for each gene targeted by a probe. This was the per-gene summary statistic used as the response variable **z** in the linear model described above.

## Results and discussion

The complete data are shown in Additional file 3.

### Consideration of pathway overlaps

**Figure 1:**
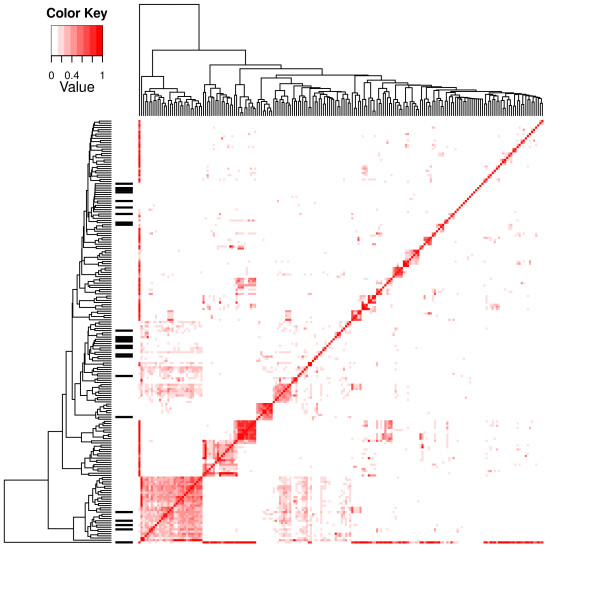
** Dendrogram of the relative overlaps among all KEGG pathways calculated as the number of overlapping genes divided by the smaller of the two sets.** The black bars to the left show the pathways with a posterior probability larger than 0.99. The names of the KEGG pathways are not shown.

**Figure 2:**
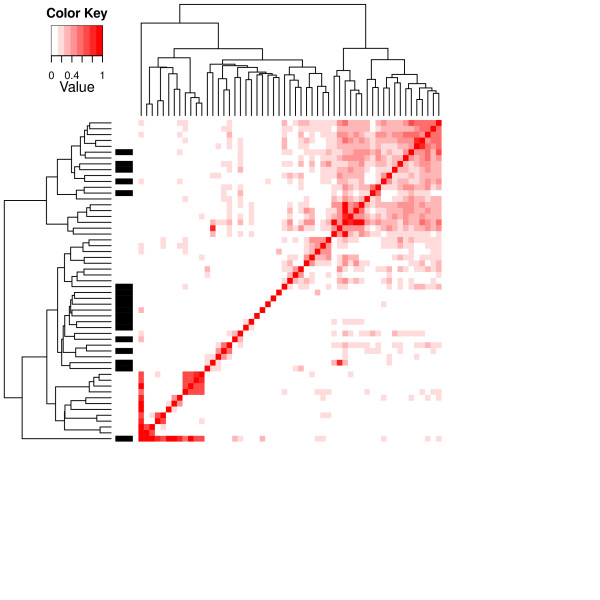
** Dendrogram showing the relative overlaps among the KEGG pathways calculated as the number of overlapping genes divided by the smaller of the two sets.** Only the KEGG pathways with a *p*-value less than 0.000001, adjusted for multiple testing by the method of Benjamini and Yekutieli [12], are shown. The black bars on the left-hand side show the pathways that had a Bayes factor larger than 100.

The relative overlaps among the pathways were calculated by dividing the number of overlapping genes by the size of the smaller of the two overlapping pathways, to test whether they affect the outcome of the variable selection procedure. The heat-map in Figure 1 shows the overlaps among the pathways and the highly ranked KEGG pathways. Many of the high-ranking pathways were among the less-overlapping pathways. In the clusters of highly overlapping pathways, there were only one or a few high-ranking pathways or even none in some cases. This is expected because the Bayesian variable selection procedure considers overlaps. If the method did not consider the overlaps among pathways there would be cases of highly overlapping pathways being highly ranked by the selection procedure at the same time if many of the overlapping genes were differentially expressed. When using the ANOVA method that does not take overlaps into account, there were several gene sets that were highly overlapping among the most highly ranked gene sets (Figure 2).

**Figure 3:**
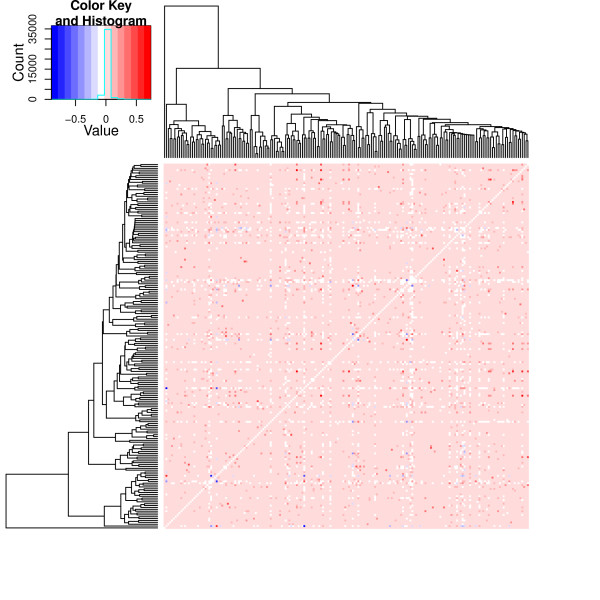
** Heat-map showing the dendrogram from the overlaps among all KEGG pathways; the points represent posterior Pearson correlations between the indicator variables, generated by Gibbs sampling, corresponding to all the KEGG pathways.** The latent variables could be either one or zero, indicating that a particular KEGG pathway is included in or excluded from the model, respectively. A positive posterior correlation would indicate pathways that are selected together in the model; and a negative correlation would indicate pathways that tend to be included in the model alternately. Ending to only select one of them at the time but not the other one or select the second one but not the first one.

The KEGG pathways were also connected to their latent variables containing information about whether the pathway was included in the Gibbs sampling round. The Pearson correlations between the latent variables were computed. A positive posterior correlation would indicate pathways that are selected together in the model; and a negative correlation would indicate pathways that tend to be included in the model alternately. These correlations for each pathway are shown in Figure 3, while the dendrogram is based on clustering according to their relative overlaps in Figure 1. The correlations do not, in general, follow a particular pattern. The highly correlated pathways are not clustered together in a pattern similar to that shown in Figure 1. This shows that the overlapping pathways are not selected together.

### Influence of prior probability on the latent variables

**Figure 4:**
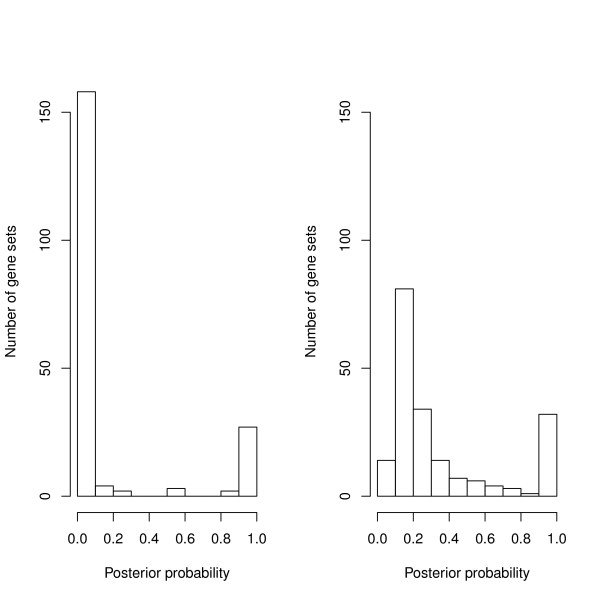
Two histograms show the number of pathways with different posterior probabilities of being included in the model (0.05 on the left and 0.40 on the right).

We assessed the robustness of the selection procedure to different levels of prior probabilities for the latent variable by increasing the probability from 0.05 to 0.40, which corresponds to including more pathways in each step of the Gibbs sampling. This resulted in more pathways with a posterior probability between the extremes of zero and one (Figure 4), but the most highly ranked pathways did not change. The 30 most highly ranking pathways were identical for the two prior probabilities. This shows that variable selection for this particular data-set is robust to a change in prior probability. However, we recommend that the influence of the prior probability be assessed for each data-set. This sensitivity analysis can also be useful because there are potential mixing problems of the sampler, as SSVS is known to suffer from slow mixing if the prior probability of the latent variable is too small [14].

### Comparison to the ANOVA approach

We compared the results from the Bayesian variable selection procedure to those of an ANOVA approach that ignores the overlaps among gene sets. The KEGG pathways highly ranked by the ANOVA approach are shown in Figure 2. From this heat-map it appears that the ANOVA approach ranks highly overlapping pathways together. Another way of investigating this tendency is that if the overlapping gene sets tend to be ranked similarly, the differences among the ranking scores would be negatively correlated to the relative overlaps. If the gene sets that are highly overlapping do not tend to have a similar score, the correlation would be zero or close to zero. For the ANOVA method, the *p*-values were used as scores and the Spearman correlation was −0.34. For the variable selection method, the Spearman correlation was −0.0063. It appears that the ANOVA approach tends to rank overlapping gene sets similarly while the Bayesian variable selection approach does not.

One illustrative example is the ‘Focal Adhesion’ pathway that contains 152 genes. It was considered highly significant by the ANOVA approach. Using the Bayesian variable selection procedure, however, it had a very low posterior probability (0.013) of being included in the model. Therefore, it is unlikely to play an important role in the acute phase response in the udder. The discrepancy can be explained because it has a large overlap (50 genes) with the ‘Extracellular Matrix (ECM)-receptor Interaction’ pathway, which is highly ranked by the variable selection method. In total, ‘ECM-receptor Interaction’ contains 63 genes. By the Bayesian method, the ‘Focal Adhesion’ pathway does not contribute greatly once the ‘ECM-receptor Interaction’ pathway is taken into account. The overlap between ‘Focal Adhesion’ and ‘Extracellular Matrix (ECM)-receptor Interaction’ is shown in a Venn diagram in Additional file 4.

### Cases of highly overlapping pathways

When the genes are highly overlapping in two pathways it can be difficult for the Bayesian variable selection procedure to select one of them. Consider for example the following two simple pathways: A- > B- > C and B- > C- > D. These pathways overlap by genes B and C, and it would be hard to distinguish the more influential pathway if genes B and C were highly expressed. In the variable selection procedure, one of the pathways would be included in the model interchangeably. This is reflected in the posterior correlation for the latent variables. If the two pathways were interchangeably included in the model the correlation would be negative. We searched for examples of pathways with a high overlap and a negative posterior correlation. For both prior probabilities for the latent variable, the posterior correlations were centered on zero with the majority (95–98%) of the posterior samples being between −0.1 and 0.1.

When using a prior probability of 0.05 of including a pathway in the model, there were only two examples of low posterior correlation below −0.1 and a large overlap: ‘Tight Junction’ and ‘Leukocyte Transendothelial Migration’. The correlation was −0.15 and the relative overlap was 0.43. This was not an example of a pair of pathways that were hard to discriminate, because ‘Tight Junction’ had a high posterior probability (0.94) and ‘Leukocyte Transendothelial Migration’ had a low posterior probability (0.013).

When using a prior probability of including the pathways in the model of 0.4, the two pathways ‘Huntington’s Disease’ and ‘Parkinson’s Disease’ were noteworthy. These two pathways are both involved in neurodegenerative diseases and have a negative posterior correlation of −0.37. Their relative overlap was 0.81. Neither pathway had a much higher posterior than prior probability of being included in the model. However, both were highly ranked by the ANOVA method. This provides an example of two pathways that are hard to discriminate by the Bayesian variable selection method. On the other hand, it also illustrates a feature of the variable selection procedure that permits a deeper insight into biologically relevant expression patterns in the data.

### The top-ranked pathways

The 30 top-ranked pathways are shown in Table 1. These top-ranked pathways are primarily characterized by activation of the immune response. The response is manifested by 1) lysis of bacteria and cells *via* lysosome, complement, and coagulation cascades and Fc gamma R-mediated phagocytosis; 2) leukocyte migration and inflammation indicated by cell adhesion molecules and chemokine signaling pathways; and 3) cell differentiation and proliferation led by ECM-receptor interactions, cytokine-cytokine receptor interactions, and the MAPK signaling pathway. Another important observation regarding the highly ranked pathways is the presence of several metabolic pathways such as aminoacyl-tRNA biosynthesis, purine metabolism, and ABC transporters. It is worth noting the presence of signaling pathways that regulate a variety of cellular functions, including inflammation and metabolism. For example, the PPAR signaling pathway regulates ABC lipid transporters and is a molecular link between the immune system and macronutrient metabolism [21].

**Table 1:** 30 top-ranked KEGG pathways using a prior probability of 0.05

**KEGG pathway**	**Number of genes**	**Posterior probability of being included in the model**	**Odds ratio**	**Variance per gene (10^-3)**
ABC transporters	24	1	Infinity	4.24
Lysosome	90	1	Infinity	3.56
Proteasome	40	1	Infinity	3.53
Complement and coagulation cascades	53	1	Infinity	3.13
RIG-I-like receptor signaling pathway	44	1	Infinity	2.80
ECM-receptor interaction	63	1	Infinity	2.59
Cell adhesion molecules (CAMs)	95	1	Infinity	2.50
Axon guidance	79	1	Infinity	2.46
SNARE interactions in vesicular transport	31	1	Infinity	2.39
RNA degradation	49	1	Infinity	2.39
Ubiquitin mediated proteolysis	105	1	Infinity	2.34
Neuroactive ligand-receptor interaction	125	1	Infinity	2.27
PPAR signaling pathway	58	1	Infinity	2.16
Ribosome	76	1	Infinity	2.15
MAPK signaling pathway	179	1	Infinity	2.11
Aminoacyl-tRNA biosynthesis	34	1	Infinity	2.08
Endocytosis	139	1	Infinity	2.08
Fc gamma R-mediated phagocytosis	70	1	Infinity	2.00
Insulin signaling pathway	97	1	Infinity	1.88
Cell cycle	96	1	Infinity	1.85
Notch signaling pathway	31	1	Infinity	1.73
Cytokine-cytokine receptor interaction	115	1	Infinity	1.69
Chemokine signaling pathway	138	1	Infinity	1.58
Metabolic pathways	793	1	Infinity	1.47
Tight junction	97	0.938	288	1.11
Purine metabolism	117	0.927	240	1.16
Chronic myeloid leukemia	58	0.919	215	1.27
Pathways in cancer	227	0.847	105	0.945
Basal transcription factors	23	0.826	90.3	1.28
Circadian rhythm - mammal	5	0.590	27.4	1.77

“Odds ratio” indicates the odds ratio between the prior and posterior probability of being included in the model. “Variance per gene” means the estimated variance of the *t*-statistic per gene. The gene sets are ranked primarily according to the posterior probabilities and secondarily according to the variance per gene.

As shown in Table 1, a potential limitation is that it was not possible to discriminate among the 24 most highly ranked gene sets in terms of significance using the posterior probability of being included in the model or the odds ratio between the prior and posterior probabilities. To distinguish them, it may be helpful to study the variance explained per gene by each of the sets. ‘ABC Transporters’ and ‘Lysosome’ were the two most highly ranked gene sets using this way of ranking. However, neither of these was identified in a hypergeometric gene set enrichment study on gene sets defined by KEGG pathways taking only the differentially expressed genes [16].

### Robustness to a limited number of observations

Our gene set approach uses a moderated *t*-statistic, the per-gene summary statistic, as the response variable. The summary statistics were computed for each of the 3130 genes linked to KEGG pathways, but were based on a limited number of observations—only eight animals in each treatment group in this study. We assessed the influence of a limited number of observations on the robustness of the high-ranking pathways by randomly selecting a subset of the animals, computing the moderated *t*-statistics, and running the variable selection procedure. We repeated this 120 times, eliminating the data from two animals at a time, and recorded the rankings of the pathways in each round. Among the 30 highest-ranking pathways in each round, 26 pathways appeared in all 120 runs. Although these results suggest that our approach is robust in cases where there is a limited number of observations, we suggest that this type of analysis should be performed for each data-set.

## Conclusions

Bayesian variable selection can prioritize gene sets while also considering the overlaps among them. This can be performed for a large number of genes without overwhelmingly demanding computation. The selection method tends to select one or a few pathways among the highly overlapping pathways. It also makes it possible to determine which pairs of overlapping pathways are harder to prioritize. This can be achieved by studying the latent variables computed in the variable selection.

Our results show that the ANOVA approach can give misleading results by not considering the pathway overlaps. The Bayesian variable selection method gave similar results to the ANOVA method, but it was able to highlight one or a few among the highly overlapping genes.

Cases that would prove difficult for the Bayesian variable selection method include when there are very highly overlapping genes and the overlapping genes are differentially expressed. In such cases, we suggest that the posterior correlations among highly overlapping pathways should be examined to determine whether they are negative and have a high absolute value.

## Abbreviations

AIC, Akaike’s information criterion; ANOVA, Analysis of variance; ECM, Extracellular matrix; GO, Gene ontology; GSEA, Gene set enrichment analysis; KEGG, Kyoto encyclopedia of genes and genomes; SSVS, Stochastic search variable selection.

## Competing interests

The authors declare that they have no competing interests.

## Authors’ contributions

AS and PS conceived and designed the project. AS analyzed the data assisted by PS, MS, and LUJ (Luc). All authors drafted the manuscript and contributed to the interpretation of the results. All authors have read and approved the final manuscript.

## Supplementary Material

Additional file 1 **Matrix where the rows represent the genes with their Entrez gene identifiers.** The columns represent the Kyoto Encyclopedia of Genes and Genomes pathways. The elements can have the values zero or one. The value zero means that the gene is not in the pathway whereas the value one means that the gene is in the pathway. Click here for file

Additional file 2 Details of the Bayesian analysis. Click here for file

Additional file 3 **Table showing the full list of gene sets, defined according to KEGG pathways, ranked according to the variable selection method. “Odds_ratio” means the odds ratio between the prior and posterior probability for the gene set of being included in the model.** This is used as a Bayes factor to judge the significance. “Inf” indicates infinity, which is the evaluated Bayes Factor when the posterior probability is 1. “Explained variance” is the average variance explained by the *t*-statistic per gene in the gene set. Click here for file

Additional file 4 **The Venn diagram is showing the overlap of genes between the pathways ‘Focal Adhesion’ and ‘Extracellular Matrix (ECM)-receptor Interaction’.** ‘Focal adhesion’ was highly ranked by the ANOVA method but had a low posterior probability (0.013) of being included in the model when using the Bayesian method. A plausible reason for this is the high overlap with the pathway ‘Extracellular Matrix (ECM)-receptor Interaction’, which had a posterior probability 1 of being included in the model. These results indicate that ‘Focal Adhesion’ was ranked high in the ANOVA due to ‘guilt by association’ with the ‘ECM-receptor interaction’ pathway. Click here for file
