# Supplementary material for: A Bayesian variable selection procedure to rank overlapping gene sets
Source: BMC Bioinformatics. 2012 May 3;13:73. doi: 10.1186/1471-2105-13-73 (PMC3434019; doi:10.1186/1471-2105-13-73)
Supplement: Additional file 2 — Details of the Bayesian analysis. [file 1471-2105-13-73-S2.pdf]

APPENDIX
DETAILS OF BAYESIAN ANALYSIS

This Appendix describes the details of the Bayesian analysis using a Stochastic Search Variable Selection (SSVS) to select gene sets. The SSVS is based on George & McCulloch (1993) who proposed the use of a mixture of Normal densities as a prior distribution in a hierarchical Bayesian model as an approach for a model selection algorithm. We extended and modified the George & McCulloch (1993) implementation by adding a model intercept, by taking the "large" variance in the mixture as a model parameter to be learnt from the data, and by choosing for uniform prior distributions on variance parameters. This section describes the Bayesian posterior density for this model and the conditional distributions needed to implement a Markov chain Monte Carlo (MCMC) algorithm to obtain samples from the posterior distribution of the parameters in this model.

The complete Bayesian model specification is as follows:

$$
\begin{aligned}
(1) \qquad z =& 1\mu + X\beta + e \\
&\mu \sim U(-\infty, \infty) \\
&\beta_i \sim (1-\gamma_i)N(0,\tau_0^2) + \gamma_i N(0,\tau_1^2) \\
&\gamma_i \sim Bern(\pi) \\
&e \sim N(0, I\sigma^2) \\
&\tau_1^2, \sigma^2 \sim U(0,\infty)
\end{aligned}
$$

where z are the gene statistics used as responses (length n), as explained in the main text, μ is an intercept, X is a covariate/classification matrix with rows for genes and columns for gene sets and containing 0's and 1's to indicate membership of genes to sets (genes can belong to multiple sets causing overlapping gene sets), β are regression coefficients, and e residual model errors. In the distributional assumption, $U()$ indicates a Uniform distribution with left bound and right bound, $N()$ a Normal with mean and variance, and $Bern()$ a Bernoulli with probability for being 1. The Bayesian model specifies that μ has a uniform prior distribution, causing the intercept to be fitted as "fixed" (unshrunken), β is modeled using a mixture, and residuals are assumed Normal. The fitting of the mixture is using the auxiliary Bernoulli variables γ_i, indicating whether β_i is in the first ($\gamma_i = 0$) or second mixture ($\gamma_i = 1$). The posterior mean of γ_i is also used as the posterior probability that the ith covariate is selected in the model. In the mixture distribution, τ_1^2 (slab variance) is taken as a model parameter and is learned from the data, using a Uniform prior. The parameters τ_0^2 and π are taken as known values. The τ_0^2 was set to 0.027, which is a value such that all 'out of model'

covariates collectively do not explain more than 1% of variance in the responses. The proportion of expected important gene sets π was set to 0.05. A sensitivity analysis was performed on the selection of the gene sets by increasing π to 0.40. Also the variance of residuals σ^2 is taken as a model parameter and is learned from the data using a Uniform prior.

The joint posterior distribution (apart from constants) for the above model is:

$$(2)\qquad f(\mu,\beta,\gamma,\tau_1^2,\sigma^2|z) \propto \\ (\sigma^2)^{-n/2}\exp(-(z-1\mu-X\beta)'(z-1\mu-X\beta)/2\sigma^2) \\ \prod_i(\tau_{\gamma_i}^2)^{-1/2}\exp(-\beta_i^2/2\tau_{\gamma_i}^2)\prod_i(1-\pi)^{(1-\gamma_i)}\pi^{\gamma_i}$$

where the first expression is the likelihood, the second is the mixture prior for the β_i's, and the third is the Bernoulli prior for the γ_i's. The Uniform priors used for μ,τ_1^2,σ^2 are not explicitly shown because they add a constant which disappears in the proportional expression of the posterior used here.

The MCMC algorithm to obtain samples from the posterior distribution of the model parameters cycles through sampling each parameter from its conditional posterior distribution given the other parameters and the data. These conditional distributions can be identified by removing all parts from the posterior that do not depend on the parameter of interest (are multiplying constants), and performing algebra until a distributional form can be recognized.

The conditional posterior distribution for the model intercept μ is:

$$(3)\qquad f(\mu|\beta,\gamma,\tau_1^2,\sigma^2,z) \propto \\ (\sigma^2)^{-n/2}\exp(-(z-1\mu-X\beta)'(z-1\mu-X\beta)/2\sigma^2)$$

where using some algebra and expressing $\tilde{z}=z-X\beta$ gives:

$$(4)\qquad f(\mu|\beta,\gamma,\tau_1^2,\sigma^2,z) \propto \\ (\sigma^2)^{-n/2}\exp(-(\mu-(1'1)^{-1}1'\tilde{z})'(1'1)(\mu-(1'1)^{-1}1'\tilde{z})/2\sigma^2)$$

which shows that the conditional distribution of the model intercept is Normal with mean $(1'1)^{-1}1'\tilde{z}$ and variance $(1'1)^{-1}\sigma^2$. Note that $(1'1)$ is simply the length n of z.

For the regressions coefficients β we use single-variate Gibbs updates, i.e., update every β_i given all other β_j and other relevant parameters and data. For an SSVS approach this scheme is more efficient than a joint updating scheme, because in the SSVS all regression coefficient are non-zero and this is often a (very) large set of regression coefficients. The conditional posterior distribution for one regression

coefficient β_i is:

$$(5)\qquad f(\beta_i|\mu,\beta_j,\gamma,\tau_1^2,\sigma^2,z) \propto \\ (\sigma^2)^{-n/2}\exp(-(z-1\mu-x_i\beta_i-X_j\beta_j)'(z-1\mu-x_i\beta_i-X_j\beta_j)/2\sigma^2) \\ (\tau_{\gamma_i}^2)^{-1/2}\exp(-\beta_i^2/2\tau_{\gamma_i}^2)$$

Here we use $\tilde{z} = z - 1\mu - X_j\beta_j$, and with some algebra obtain:

$$(6)\qquad f(\beta_i|\mu,\beta_j,\gamma,\tau_1^2,\sigma^2,z) \propto \\ (\sigma^2)^{-n/2}\exp(-(\beta_i-(x_i'x_i)^{-1}x_i'\tilde{z})'(x_i'x_i)(\beta_i-(x_i'x_i)^{-1}x_i'\tilde{z})/2\sigma^2) \\ (\tau_{\gamma_i}^2)^{-1/2}\exp(-\beta_i^2/2\tau_{\gamma_i}^2)$$

These are two kernels of Normal densities for β_i that can be combined using standard text book results to give the conditional distribution of β_i to have mean $(x_i'x_i+\sigma^2/\tau_{\gamma_i}^2)^{-1}x_i'\tilde{z}$ and variance $(x_i'x_i+\sigma^2/\tau_{\gamma_i}^2)^{-1}\sigma^2$. This is a common expression for a "random" effect fit of β_i with shrinkage selected by the γ_i indicator variable to be either strong ($\gamma_i = 0, \tau_0^2$ small) or mild ($\gamma_i = 1, \tau_1^2$ larger).

The indicator variable γ_i is a binary parameter for which it is convenient to directly compute the ratio between the probabilities $Pr(\gamma_i = 1)/Pr(\gamma_i = 0)$ in the posterior distribution. In this ratio, everything cancels which does not depend on γ_i, leaving:

$$(7)\qquad \frac{Pr(\gamma_i=1|\mu,\beta,\gamma_j,\tau_1^2,\sigma^2,z)}{Pr(\gamma_i=0|\mu,\beta,\gamma_j,\tau_1^2,\sigma^2,z)} = \frac{(\tau_1^2)^{-1/2}\exp(-\beta_i^2/\tau_1^2)\pi}{(\tau_0^2)^{-1/2}\exp(-\beta_i^2/\tau_0^2)(1-\pi)}$$

A new γ_i can be sampled by drawing a uniform random deviate $u \sim U(0,1)$, and set the indicator variable to 1 if the ratio $u/(1-u)$ is smaller than the above ratio, or to 0 otherwise.

The conditional posterior distribution of the slab variance τ_1^2 depends only on those β_i that, for a particular MCMC cycle, are selected to be "large" by having $\gamma_i = 1$. Let the set of "large" β's be denoted by S_L, with n_L members, then the relevant part from the posterior for the conditional distribution of τ_1^2 is:

$$(8)\qquad f(\tau_1^2|\mu,\beta,\gamma,\sigma^2,z) \propto \prod_{i\in S_L}(\tau_1^2)^{-1/2}\exp\left(-\beta_i^2/2\tau_1^2\right) \\ \propto (\tau_1^2)^{-n_L/2}\exp\left(-\sum_{i\in S_L}\beta_i^2/2\tau_1^2\right)$$

This is a scaled inverse chi-square with $n_L - 2$ degrees of freedom and shape parameter $(\sum_{i\in S_L}\beta_i^2)/(n_L-2)$.

For the conditional posterior distribution of the model residual variance σ^2, we use $\tilde{z} = z - 1\mu - X\beta$ (the model residuals), so that the conditional posterior can

be expressed as:

(9) $$f(\sigma^2|\mu, \beta, \gamma, \tau_1^2, z) \propto (\sigma^2)^{(-n/2)} \exp(-\tilde{z}'\tilde{z}/2\sigma^2)$$

This is a scaled inverse chi-square with $n-2$ degrees of freedom and shape parameter $\tilde{z}'\tilde{z}/(n-2)$.
