# Supplementary figures and images for: A Bayesian variable selection procedure to rank overlapping gene sets
Source: BMC Bioinformatics. 2012 May 3;13:73. doi: 10.1186/1471-2105-13-73 (PMC3434019; doi:10.1186/1471-2105-13-73)

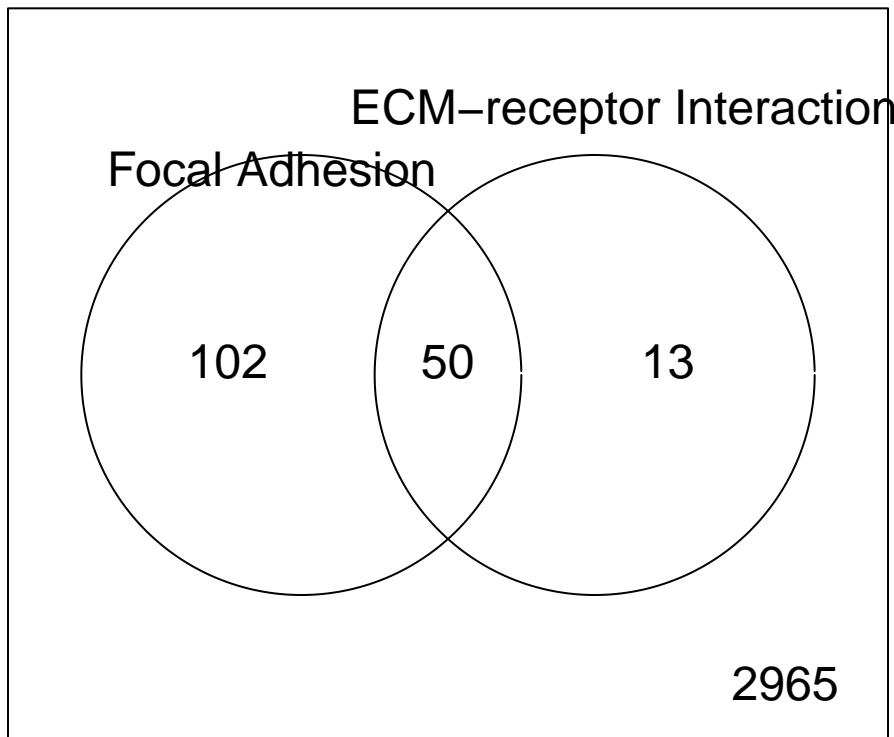

Supplement: Additional file 4 — The Venn diagram is showing the overlap of genes between the pathways ‘Focal Adhesion’ and ‘Extracellular Matrix (ECM)-receptor Interaction’. ‘Focal adhesion’ was highly ranked by the ANOVA method but had a low posterior probability (0.013) of being included in the model when using the Bayesian method. A plausible reason for this is the high overlap with the pathway ‘Extracellular Matrix (ECM)-receptor Interaction’, which had a posterior probability 1 of being included in the model. These results indicate that ‘Focal Adhesion’ was ranked high in the ANOVA due to ‘guilt by association’ with the ‘ECM-receptor interaction’ pathway. [file 1471-2105-13-73-S4.pdf]
